# Supplementary material for: Epidemiology and demographics of slipped capital femoral epiphysis in South Korea: A nationwide cohort study
Source: PLoS One. 2023 Mar 31;18(3):e0283123. doi: 10.1371/journal.pone.0283123 (PMC10065226; doi:10.1371/journal.pone.0283123)
Supplement: Attachment — Submitted filename: WYFRP_17_Revision_220622_MJJ_0709edit.docx [file pone.0283123.s001.docx]

PONE-D-21-25828

Epidemiology and demographics of slipped capital femoral epiphysis in South Korea: a nationwide cohort study

PLOS ONE

Reviewers' comments:

Reviewer's Responses to Questions

Comments to the Author

1. Is the manuscript technically sound, and do the data support the conclusions?

The manuscript must describe a technically sound piece of scientific research with data that supports the conclusions. Experiments must have been conducted rigorously, with appropriate controls, replication, and sample sizes. The conclusions must be drawn appropriately based on the data presented.

Reviewer #1: Yes

2. Has the statistical analysis been performed appropriately and rigorously?

Reviewer #1: Yes

3. Have the authors made all data underlying the findings in their manuscript fully available?

The [PLOS Data policy](http://www.plosone.org/static/policies.action#sharing) requires authors to make all data underlying the findings described in their manuscript fully available without restriction, with rare exception (please refer to the Data Availability Statement in the manuscript PDF file). The data should be provided as part of the manuscript or its supporting information, or deposited to a public repository. For example, in addition to summary statistics, the data points behind means, medians and variance measures should be available. If there are restrictions on publicly sharing data—e.g. participant privacy or use of data from a third party—those must be specified.

Reviewer #1: Yes

4. Is the manuscript presented in an intelligible fashion and written in standard English?

PLOS ONE does not copyedit accepted manuscripts, so the language in submitted articles must be clear, correct, and unambiguous. Any typographical or grammatical errors should be corrected at revision, so please note any specific errors here.

Reviewer #1: Yes

5. Review Comments to the Author

Please use the space provided to explain your answers to the questions above. You may also include additional comments for the author, including concerns about dual publication, research ethics, or publication ethics. (Please upload your review as an attachment if it exceeds 20,000 characters)

Reviewer #1: A very nice study. I have some comments that I believe will strengthen the study.

**Thank you for your time and effort in reviewing this paper. We have made the appropriate changes to the manuscript.**

Line 42 – SCFE is not truly a separation except in the unstable type. The more common stable type is just a shift in position of the epiphysis to the metaphysis. Please reword this.

**Thank you for the comment. We have reworded this definition in the manuscript as follows:**

**“Slipped capital femoral epiphysis (SCFE) is associated with many complications and deformities secondary to a shift in position of the epiphysis in relation to the metaphysis of the proximal femur.”**

**“When combined with increased body weight and retroversion of the femoral head, the loads may exceed the ultimate yield point, causing a shift in position of the epiphysis in relation to the metaphysis of the femoral head. In severe cases, which are classified as unstable, a true separation may occur.”**

Line 47 – causing the epiphysis to slip relative to the metaphysis – it is not the femoral that head that is slipping, as the head included both the epiphysis and the proximal metaphysis.

**Thank you for your comment. We have made the following changes:**

**“When combined with increased body weight and retroversion of the femoral head, the loads may exceed the ultimate yield point, causing a shift in position of the epiphysis in relation to the metaphysis of the femoral head. In severe cases, which are classified as unstable, a true separation may occur.”**

Methods

Are all Korean patients in the HIRA database, or only those with government insurance. I am sorry I don’t know how Korean insurance works – are there private insurance companies as well as a national health insurance. I think American readers would want to know how this works.
**Thank you for this comment. The Korean healthcare is a national health insurance, and all medical costs are covered by the national insurance system. It would be rational to assume that all Korean patients are in the HIRA database. We have clarified this point in the manuscript as following:**

**“All Korean patients are enlisted in HIRA, as the Korean healthcare is a national health insurance.”**

Also, a general overall question – how accurate is the HIRA database – are there some cases that would be missed, or secondary information for a SCFE case (eg endocrine disorders, etc). I think this should be mentioned in the Material and Methods section. Have accuracy studies been done?

**All patients in the HIRA database are enlisted in accordance with diagnoses made by their doctors, and the records are stored electronically. Diseases that do not require a specialist opinion for diagnosis, such as the common cold, may be less accurately reflected in the database. On the other hand, diseases such as SCFE that require a pediatric orthopedic board-certified doctor for diagnosis are very accurately reflected in the HIRA database. Although a separate accuracy study has not been conducted, we believe that the process of patient registration guarantees the accuracy of the HIRA database regarding the current study on SCFE.**

**We have clarified this point in the Material and Methods section as follows:**

“Nearly all information on patients and their medical records can be obtained from this database, which has been used in previous epidemiology studies. **Accuracy of the database regarding SCFE is guaranteed as patients diagnosed with SCFE are registered in HIRA upon diagnosis made by pediatric orthopedic specialists.”**

Results
Line 111 instead of using the word comorbidity, I would use disease, as done in the same sentence with pituitary disease.

**Thank you for your comment. We have made the relevant changes.**

Line 116-117 – can this be further explained? Were the 15 cases of same day surgery due to simultaneous bilateral SCFE, and the 64 for patients that developed a 2nd sequential SCFE later on?

**This is correct. Of the 79 patients who were recorded in the HIRA database as having undergone bilateral hip operations, 15 have undergone bilateral in-situ pinning on the same day. Bilateral in-situ pinning was done with some time interval in between in the remaining 64 patients, possibly due to second sequential SCFE. However, whether bilateral in-situ pinning was done simultaneously because of bilateral SCFE or for prophylactic purposes cannot be determined with data extracted from the HIRA database. We have clarified this in the discussion and included it as a limitation:**

**Discussion:**

**“In our study, 79 patients (13.5%) underwent bilateral hip surgery. In 15 cases, bilateral surgery was performed on the same day; however, whether these procedures were done for prophylactic purposes or for treatment of bilateral SCFE cannot be ascertained given the nature of the HIRA database.”**

**Limitations:**

**“**This study has some limitations, which stem mainly from its retrospective cohort design. First, we could not account for misclassifications, such as incorrect billing or wrong diagnostic codes. However, only patients with a diagnosis of SCFE who underwent related surgery within 90 days were included in the study, which we believe minimized the likelihood of the initial diagnosis being incorrect. Second, we could not test for a correlation between the incidence of SCFE and obesity because the HIRA database does not include information on BMI. **Third, it is difficult to discern the reason for simultaneous bilateral hip surgeries with the HIRA database. We cannot know whether simultaneous bilateral hip surgeries were done for prophylactic purposes or for treatment of bilateral SCFE, for the database lacks information on laterality.**”

Line 119 – Was the femoral osteotomy the treatment for the SCFE or for the AVN; same question for the pelvic osteotomy and total hip arthroplasty.

**Femoral, pelvic osteotomy and total hip arthroplasty were done for treatment of AVN. We have further clarified as follows:**

**“**Avascular necrosis occurred at a mean of 26.4 months after the initial surgery for SCFE in 25 patients. **Twelve patients with AVN have undergone femoral osteotomy, with one case each of pelvic osteotomy and total hip arthroplasty.**”

Do we know why AVN developed? Was it because they were unstable SCFEs or was it due to treatment of the SCFE (eg modified Dunn procedure, etc)? Please amplify. If not known because it wasn’t in the data base please state so.

**The etiology of AVN after SCFE cannot be discerned with information from the HIRA database. We have added this to the limitations:**

“This study has some limitations, which stem mainly from its retrospective cohort design. First, we could not account for misclassifications, such as incorrect billing or wrong diagnostic codes. However, only patients with a diagnosis of SCFE who underwent related surgery within 90 days were included in the study, which we believe minimized the likelihood of the initial diagnosis being incorrect. Second, we could not test for a correlation between the incidence of SCFE and obesity because the HIRA database does not include information on BMI. Third, it is difficult to discern the reason for simultaneous bilateral hip surgeries with the HIRA database. We cannot know whether simultaneous bilateral hip surgeries were done for prophylactic purposes or for treatment of bilateral SCFE, for the database lacks information on laterality. **Fourth, the type of SCFE cannot be distinguished with the HIRA database. Therefore, whether AVN after SCFE is the result of treatment or the disease itself cannot be determined.**”

Line 143 – delete “detectable” it was obviously an increase!

**We deleted the word. Thank you for your comment.**

Line 154 and Figure 4 – are these data for Korea? I assume so, but please state, and include in the legend for Figure 4.

**We clarified the legend to state that the data is from a Korean population:**

“Figure 4. Annual increase in obesity rate based on body mass index (BMI) since 2009 in the **Korean** population aged 9–14 years.”

Lines 176-178 – how can it be assumed the 15 bilateral cases were for prophylactic fixation? If it is not stated in the HIRA database just state that – certainly simultaneous bilateral SCFEs exist, and in the US account for about 50% of bilateral cases.

**Thank you for the comment. With the HIRA database, the reason for simultaneous bilateral cases cannot be discerned. We have made the relevant corrections as follows:**

**“In our study, 79 patients (13.5%) underwent bilateral hip surgery. In 15 cases, bilateral surgery was performed on the same day; however, whether these procedures were done for prophylactic purposes or for treatment of bilateral SCFE cannot be ascertained given the innate nature of the HIRA database.”**

**We have also included this shortcoming in the limitations section:**

**“**This study has some limitations, which stem mainly from its retrospective cohort design. First, we could not account for misclassifications, such as incorrect billing or wrong diagnostic codes. However, only patients with a diagnosis of SCFE who underwent related surgery within 90 days were included in the study, which we believe minimized the likelihood of the initial diagnosis being incorrect. Second, we could not test for a correlation between the incidence of SCFE and obesity because the HIRA database does not include information on BMI. **Third, it is difficult to discern the reason for simultaneous bilateral hip surgeries with the HIRA database. We cannot know whether simultaneous bilateral hip surgeries were done for prophylactic purposes or for treatment of bilateral SCFE, for the database lacks information on laterality.**”

Discussion:
Lines 191-192 - this answers one of my previous questions – but please state it earlier so that the reader is now wondering until the last paragraph.

**Thank you for your comment. We have clarified the figure legend to more clearly state that this is an epidemiologic study based on the Korean population.**

Table 1: What is meant by estrogen therapy? Why was it given? For what disease? As is known, estrogen causes physeal closure and tends to be protective against SCFE (thus why less common in adolescent girls than boys). Thus can we be sure that the estrogen therapy was causative for the SCFE or just an association?

**In cases of delayed puberty, sex hormone therapies can be used for treatment. In case of pituitary insufficiencies, when there are deficiencies in excretion of both growth hormone and sex hormones, estrogen and growth hormone can be administered simultaneously for treatment of short stature and delayed puberty. We assume that the two cases of estrogen therapy are those who were simultaneously administered with growth hormone. Therefore, we agree with your comment that the incidence of SCFE in these two cases are the result of co-administered growth hormone rather than estrogen therapy itself. We have deleted this column from the table.**

| Table 1. Comorbidities and History of hormone and radiation therapy in SCFE patients | | | |
| --- | --- | --- | --- |
|  | Total SCFE patients  (N=586) | MALE  (N=429) | FEMALE  (N=157) |
| Comorbidity |  |  |  |
| Pituitary gland disease | 29 (4.95) | 11 (2.56) | 18 (11.46) |
| Thyroid disease | 60 (10.24) | 30 (6.99) | 30 (19.11) |
| Parathyoid disease | 12 (2.05) | 8 (1.86) | 4 (2.55) |
| Hypogonadism | 16 (2.73) | 12 (2.80) | 4 (2.55) |
| Turner syndrome | 1 (0.17) | 0 (0.00) | 1 (0.64) |
| Scurvy | 1 (0.17) | 1 (0.23) | 0 (0.00) |
| History of hormone therapy |  |  |  |
| Growth hormone | 10 (1.71) | 4 (0.93) | 6 (3.82) |
| ~~Estrogen~~ | **~~2 (0.34)~~** | **~~0 (0.00)~~** | **~~2 (1.27)~~** |
| History of radiation therapy | 11 (1.88) | 5 (1.17) | 6 (3.82) |

Figures.
Figure 1 – can be easily deleted as the information is given in the Results.
Figures 2 and 3 are nice.

**Thank you for your comment. We will remove Figure 1 to reduce redundancy.**

6. PLOS authors have the option to publish the peer review history of their article ([what does this mean?](https://journals.plos.org/plosone/s/editorial-and-peer-review-process#loc-peer-review-history)). If published, this will include your full peer review and any attached files.


If you choose “no”, your identity will remain anonymous but your review may still be made public.


Do you want your identity to be public for this peer review? For information about this choice, including consent withdrawal, please see our [Privacy Policy](https://www.plos.org/privacy-policy).

Reviewer #1: No

[NOTE: If reviewer comments were submitted as an attachment file, they will be attached to this email and accessible via the submission site. Please log into your account, locate the manuscript record, and check for the action link "View Attachments". If this link does not appear, there are no attachment files.]

While revising your submission, please upload your figure files to the Preflight Analysis and Conversion Engine (PACE) digital diagnostic tool, <https://pacev2.apexcovantage.com/>. PACE helps ensure that figures meet PLOS requirements. To use PACE, you must first register as a user. Registration is free. Then, login and navigate to the UPLOAD tab, where you will find detailed instructions on how to use the tool. If you encounter any issues or have any questions when using PACE, please email PLOS at [figures@plos.org](mailto:figures@plos.org). Please note that Supporting Information files do not need this step.
