# Supplementary material for: Epidemiology and demographics of slipped capital femoral epiphysis in South Korea: A nationwide cohort study
Source: PLoS One. 2023 Mar 31;18(3):e0283123. doi: 10.1371/journal.pone.0283123 (PMC10065226; doi:10.1371/journal.pone.0283123)
Supplement: Attachment — Submitted filename: WYFRP_19_response_to_reviewer.docx [file pone.0283123.s002.docx]

Please use the space provided to explain your answers to the questions above. You may also include additional comments for the author, including concerns about dual publication, research ethics, or publication ethics. (Please upload your review as an attachment if it exceeds 20,000 characters)

Reviewer #2: All queries and comments have adequately been attended to.
It reads well and is scientifically sound.

Reviewer #3: Authors answered all previous reviewer's concerns.

There are still, however, some points that deserve further clarification:

1) Authors should provide rate of Stable vs Unstable SCFE, and repeat analysis separately (rate of complication is different between the two groups). This separation should also be reflected in their analysis according to age. Comparison should then be made.

**Thank you for your comments. As the raw data we used for the analysis in this study are HIRA data, a separate analysis between stable and unstable SCFE cannot be performed due to the inherent nature of the HIRA database. We will include this point in the limitations section as follows.**

“**Fourth, the type of SCFE cannot be distinguished with the HIRA database. Therefore, the rate of stable and unstable SCFE, as well as whether AVN after SCFE is the result of treatment or the disease itself, cannot be determined.**”

2) Authors did not mention 'pistol grip deformity'.

**In the HIRA database, the diagnosis was based on ICD-10 terminology. In ICD-10, pistol grip deformity, also known as cam-type femoroacetabular impingement (FAI), is registered as femoroacetabular impingement. Therefore, we used the term FAI instead of pistol grip deformity. We will add a pistol grip deformity in the appropriate section of FAI.**

**“**Femoroacetabular impingement **(pistol grip deformity)** occurred at an average of 45.6 months after the initial surgery in 13 patients and chondrolysis at an average of 9.3 months in two patients.**”**

3) How AVN was assessed?

**As mentioned in the text, the AVN was assessed according to its diagnostic code in the HIRA database.**

“**Avascular necrosis, femoroacetabular impingement, and chondrolysis were identified by their diagnostic codes alone.”**

4) Patient flowchart is missing (and it should include the distinction between ambulatory and non-ambulatory patients).

**Thank you for your comments. Because the data we used were retrieved from the HIRA database, it is not feasible to create a patient flow chart. For the same reason, distinguishing between ambulatory and non-ambulatory patients is also not feasible.**

5) More discussion about changing trend in obesity (it looks the rate of younger patients with obesity is increasing and getting closer to older patients). Is the rate of SCFE reflected by this trend? Is the proportion of SCFE the same at all ages (year by year, please, for this particular aspect. This should be discussed +++. Please implement Discussion.

**Thank you for your comments. The HIRA data did not reflect body mass index (BMI). Therefore, we retrieved the data for BMI and analyzed trends according to age. We postulate that the increase in the incidence of SCFE is related to an increasing trend in childhood obesity. We have included this shortcoming in the Limitations section.**

**“We could not test for a direct correlation between the incidence of SCFE and obesity because BMI was not included in the HIRA data. However, the obesity rate among Korean children and adolescents has been steadily increasing (Fig. 3) [25]. We assume that this steady increase in the rate of obesity, which is already a known risk factor for SCFE, may be an important environmental factor that influences the increase in the incidence of SCFE in Korea.**”

6) What is the importance of your findings in clinical practice? To be discussed.

**With the increase in childhood obesity, the incidence of SCFE also seems to increase. Although the prevalence of SCFE is lower in Korea than in Western countries, due to its increased incidence, SCFE should not be excluded from the differential diagnosis when an adolescent patient with hip pain is brought to the clinic. We have included this point in the conclusion as follows:**

“We could not test for a direct correlation between the incidence of SCFE and obesity because BMI was not included in the HIRA data. However, the obesity rate among Korean children and adolescents is steadily increasing (Fig. 3) [25]. We assume that this steady increase in the rate of obesity, which is already a known risk factor for SCFE, may be an important environmental factor influencing the increased incidence of SCFE in Korea.

**In the clinical setting, SCFE should be included in the differential diagnosis of adolescent patients with hip pain.**”

7) Idiopathic SCFE and SCFE with underlying pathology/secondary SCFE should be analyzed separately in terms of epidemiology and demographics and compared. Please revise.

**Thank you for your comment. As described in the Results section, 95 (16.2%) of the 586 patients had endocrine comorbidities, a history of hormone therapy, or received radiation therapy. However, the access to the raw data needed to analyze and compare idiopathic and secondary SCFE is closed and it will take at least 1 year to regain access to the database. Please understand that for such a realistic reason, the analysis is difficult under current circumstances.**
