# Supplementary material for: Epidemiology and demographics of slipped capital femoral epiphysis in South Korea: A nationwide cohort study
Source: PLoS One. 2023 Mar 31;18(3):e0283123. doi: 10.1371/journal.pone.0283123 (PMC10065226; doi:10.1371/journal.pone.0283123)
Supplement: Attachment — Submitted filename: PLOS 3rd revision_reviewer.docx [file pone.0283123.s003.docx]

Please use the space provided to explain your answers to the questions above. You may also include additional comments for the author, including concerns about dual publication, research ethics, or publication ethics. (Please upload your review as an attachment if it exceeds 20,000 characters)

Reviewer #1: All my comments have been answered. This is an excellent study and will be an addition to the SCFE epidemiology literature.

Reviewer #3: Thank you for making requested changes; the manuscript reads much better.

One additional comment:
Authors should add a Table summarizing similar studies assessing the epidemiology of SCFE, and its evolution over time, in other parts of the world, and compare percentages and type of study with yours. This should be presented in Table format.

**Thank you for your comment. We have added “table 3” to enlist and summarize epidemiology of SCFE in other countries and compared it to the results of our study.**

**Table 3. Summary of SCFE incidence as noted in previous literature**

| References | Country | Database | Duration | Patient age(yrs) | SCFE incidence | Trend | Male-to-female ratio |
| --- | --- | --- | --- | --- | --- | --- | --- |
| Herngren et al.^1^ | Sweden | NHS^*^ | 2007-2013 | 9-15 | 4.4/10,000 girls 5.7/10,000 boys | Increase in girls; constant in general population | 1.3:1 |
| Lehmann et al.^10^ | USA | 1997, 2000 KID^#^ national data | 1997, 2000 | 9-16 | 10.80/100,000 | n/a | 1.7:1 |
| Song et al.^11^ | Korea | Multicenter study | Jan 1989-Dec 2003 | 10-14 | 0.3/100,000 | n/a | 3.5:1 |
| Phadnis et al.^16^ | Maori | Regional institute | Dec 2000-Dec 2010 | 5-14 | 81/100,000 | n/a | 2:1 |
| Witbreuk et al.^17^ | The Netherlands | NHS | 1998-2010 | 5-19 | 11.6/100,000 | Increase in girls; constant in general population |  |
| Noguchi et al.^18^ | Japan | Multicenter study | Jan 1997-Dec 1999 | 0-14 | 2.2/100,000 | Rising | 5:1 |
| Perry et al.^29^ | UK | NHS | 23 years | 0-16 | 4.8/100,000 | Constant | 1.7:1 |
| Ravinsky et al.^30^ | Ontario, Canada | ICES^**^ data | Apr 2002-Mar 2011 | 0-16 | 5.7/100,000 | Decreasing | n/a |
| Tucker et al.^31^ | Northern Ireland | PACS^##^ | Jan 2013-Dec 2015 | <16 | 4.7/100,000 | Decreasing | 2:1 |
| Fedorak et al.^32^ | Samoa | Regional institute | 2005-2014 | 5-14 | 53.1/100,000 | n/a | 2:1 |
| Current study | Korea | K-HIRA data | 2007-2019 | <18 | 6/100,000 | Rising | 2.4:1 |
